# Hepatic ACAT2 Knock Down Increases ABCA1 and Modifies HDL Metabolism in Mice

**DOI:** 10.1371/journal.pone.0093552

**Published:** 2014-04-02

**Authors:** Matteo Pedrelli, Padideh Davoodpour, Chiara Degirolamo, Monica Gomaraschi, Mark Graham, Alice Ossoli, Lilian Larsson, Laura Calabresi, Jan-Åke Gustafsson, Knut R. Steffensen, Mats Eriksson, Paolo Parini

**Affiliations:** 1 Division of Clinical Chemistry, Department of Laboratory Medicine, Karolinska Institutet, Stockholm, Sweden; 2 Molecular Nutrition Unit, Department of Bioscience and Nutrition, Karolinska Institutet, Stockholm, Sweden; 3 Division of Lipid Science, Department of Pathology, Wake Forest University School of Medicine, Winston-Salem, North Carolina, United States of America; 4 Department of Pharmacological Sciences, University of Milan, Milan, Italy; 5 Cardiovascular Group, Department of Antisense Drug Discovery, Isis Pharmaceuticals, Inc., Carlsbad, California, United States of America; 6 Center for Nuclear Receptors and Cell Signaling, University of Houston, Houston, Texas, United States of America; 7 Department of Medicine, Karolinska Institute, Stockholm, Sweden; Heart Research Institute, Australia

## Abstract

**Objectives:**

ACAT2 is the exclusive cholesterol-esterifying enzyme in hepatocytes and enterocytes. Hepatic ABCA1 transfers unesterified cholesterol (UC) to apoAI, thus generating HDL. By changing the hepatic UC pool available for ABCA1, ACAT2 may affect HDL metabolism. The aim of this study was to reveal whether hepatic ACAT2 influences HDL metabolism.

**Design:**

WT and LXRα/β double knockout (DOKO) mice were fed a western-type diet for 8 weeks. Animals were i.p. injected with an antisense oligonucleotide targeted to hepatic ACAT2 (ASO6), or with an ASO control. Injections started 4 weeks after, or concomitantly with, the beginning of the diet.

**Results:**

ASO6 reduced liver cholesteryl esters, while not inducing UC accumulation. ASO6 increased hepatic ABCA1 protein independently of the diet conditions. ASO6 affected HDL lipids (increased UC) only in DOKO, while it increased apoE-containing HDL in both genotypes. In WT mice ASO6 led to the appearance of large HDL enriched in apoAI and apoE.

**Conclusions:**

The use of ASO6 revealed a new pathway by which the liver may contribute to HDL metabolism in mice. ACAT2 seems to be a hepatic player affecting the cholesterol fluxes fated to VLDL or to HDL, the latter via up-regulation of ABCA1.

## Introduction

For many years, the inhibition of intracellular cholesterol esterification has been considered as a potential strategy to prevent atherosclerosis [1]. Acyl-coenzyme A:cholesterol acyltransferase (ACAT) is an enzyme located in the endoplasmic reticulum that catalyses the synthesis of cholesteryl esters (CE) by conjugating cholesterol to long-chain fatty acids; mainly oleic and palmitic acids. It is now clear that the two enzymes ACAT1 and ACAT2, which are encoded by the *Soat1* and *Soat2* genes respectively, localize in different cell types and have separate physiological functions (for review see [2], [3]). ACAT1 is ubiquitously expressed and provides essential housekeeping functions to prevent the toxicity induced by increasing amounts of unesterified cholesterol (UC) in cells. Conversely, ACAT2 is exclusively expressed in hepatocytes and enterocytes, and it synthesizes CE that can be incorporated into apoB-containing lipoproteins (VLDL and chylomicrons). In mice deletion of *Soat1* or *Soat2* genes revealed a diverse role for the different ACAT enzymes in atherosclerosis. In *Soat1* knockout animals, ACAT1 deficiency led to a marked alteration in cholesterol metabolism resulting in massive accumulation of UC, which caused numerous skin and brain lesions, and worsened atherosclerosis. Conversely, the deletion of the *Soat2* gene has been consistently atheroprotective [4]–[6]. Previous studies also suggest a clear atherogenic potential of ACAT2-derived CE also in humans. In both women and men, the Atherosclerosis Risk in Communities (ARIC) study revealed an association between the carotid intima-media thickness and the ACAT2-derived CE in lipoproteins [7]. Also, the Uppsala Longitudinal Study of Adult Men (ULSAM) showed an association between the ACAT2-derived CE in lipoproteins and CVD mortality [8]. Finally, ACAT2-derived CE in lipoproteins were able to predict coronary artery disease in patients with symptoms of acute coronary syndrome [9]. Athero-protection was also achieved in mice using anti-sense oligonucleotide targeted to hepatic *Soat2* mRNA (ASO6) and resulting in a decreased ACAT2 activity in the liver [10]. These studies did not address whether a decreased hepatic ACAT2 activity modifies HDL metabolism. In addition, inconsistent results on HDL cholesterol levels were obtained in mice in which ACAT2 activity was reduced by genetic manipulation [4], [6], [11]–[14]. We hypothesized that a low ACAT2 activity in the liver would result in a greater UC mass that in turn could be secreted into nascent HDL by ABCA1. Hence, we reduced hepatic ACAT2 activity by injection of ASO6 in male C57BL/6 and in Liver X Receptor (LXR) α/β double knockout (DOKO) mice. In DOKO mice the link between ACAT2 activity and HDL metabolism should be more evident. This mouse model is characterized by a reduced bile acid (BA) synthesis, which is paralleled by a maintained ABCA1 expression in the liver. Considering the possible fate of cholesterol in the hepatocytes (i.e. the esterification by ACAT2 to be incorporated in VLDL or stored, the conversion into BA, and the efflux into nascent HDL) a reduction of the ACAT2 activity in DOKO mice, may indeed lead to a greater UC mass available for secretion into nascent HDL. Finally, we also investigated whether the metabolic response following a decreased hepatic ACAT2 activity was influenced by the time frame in which the animals were fed a western type of diet.

Our results identify hepatic ACAT2 as an important player for HDL metabolism since the activity of this enzyme may influence the intracellular cholesterol fated to secretion either into VLDL or into HDL particles; the latter via an increased expression of the ABCA1 protein.

## Material and Methods

### Animals and experimental design

Ten-week-old male wild-type (WT), and LXRα and LXR β double knockout (DOKO) mice, on a pure C57BL/6 genetic background (as previously described [15]), were housed on a regular 12-h light/12-h dark cycle with free access to water and food. Until the onset of the studies, mice were fed a chow diet (Special Diet Services; SDS, NOVA-SCB, Sweden – Rat and Mouse n.1 Maintenance, 9.5 mm pellet; RM1 (P)). During the two experiments animals were fed a western-type diet, containing 10% saturated fat and 0.2% cholesterol (w/w) (Harlan Laboratories - TD.07346, 4% PO, 2% SBO, 0.2% Cholesterol Diet - 1/2" pellet). In the first experiment (4-weeks), the diet was started 4 weeks prior to and maintained during the following 4 weeks of treatment with biweekly i.p. injection (25 MPK) of either an anti-sense oligonucleotide specifically targeted to hepatic ACAT2 gene (ASO 6) [10] in half of the animals, or with a control anti-sense oligonucleotide (ASOctrl) in the other half. In the second experiment (0-weeks) WT and DOKO mice were given the western-type diet for 10 weeks and concomitantly treated with the same doses of ASO6 and ASOctrl used in the 4-weeks experiment. In all the experiments, animals were fasted 4 hours prior to euthanasia, which was performed by inhalation of carbon dioxide. Blood, tissues and organs were then collected, and stored at −80°C until the analyses. The Swedish Board of Agriculture, Ethical committee on Animal Experiments, Stockholm South approved all the animal studies (Permit number: S17-07, 2007-03-30).

### Antisense Oligonucleotides (ASOs)

The 5-10-5 methoxy ethyl chimeric 20-mer oligonucleotides with fully modified phosphorothioate backbones were kindly donated by ISIS Pharmaceuticals (Carlsbad, CA, USA). ASO6 contained a sequence-targeting mouse *Soat2*. The ASOctrl l was not complementary to the ACAT2 sequence and did not hybridize with any specific gene target. The sequences of these ASOs were as follows:

ASO6: 5′-TTCGGAAATGTTGCACCTCC-3′;

ASOctrl: 5′-CCTTCCCTGAAGGTTCCTCC-3′.

### Isolation of liver microsomes and ACAT2 activity assay

Liver samples (50 to 150 mg) were homogenized in 3 mL ice-cold buffer containing 0.1 mol/L K_2_HPO_4_, 0.25 mol/L sucrose, and 1 mmol/L EDTA, pH 7.4. A protease inhibitor cocktail (Sigma) was added to the buffer before homogenization. The homogenate was then centrifuged for 15 min at 12 000 *g* (4°C) to remove cell debris. The resulting supernatant was centrifuged for 60 min at 100 000 *g*. The microsomal pellet from this spin was re-suspended in 0.1 mol/L K_2_HPO_4_ at pH 7.4 and immediately frozen at –80°C. Total ACAT enzymatic activity was determined in hepatic microsomes as previously described [16], except that pre-incubation included a cholesterol-saturated solution of β-hydroxypropyl cyclodextrin for 30 min before addition of ^14^[C] oleoyl Co-A (PerkinElmer, Uppland Väsby, Sweden). In separate tubes, pyripyropene A, a specific ACAT2 inhibitor [17], was included in the preincubation at a concentration of 5 μmol/L to differentiate ACAT1 (uninhibited) and ACAT2 (total–ACAT1) activities. Pyripyropene A was a kind gift of Prof. Hiroshi Tomoda, Dept. of Microbial Chemistry, Graduate School of Pharmaceutical Sciences, Kitasato University, Japan.

### Preparation of liver plasma membrane and western blot analysis

Liver samples (∼ 200 mg) were homogenized in 3 mL ice-cold buffer containing 20 mmol/LTris-HCl, 0.25 M sucrose, and 2 mmol/L MgCl_2_. A protease inhibitor cocktail (complete MINI Roche Diagnostics GmbH, Mannheim, Germany) was added to the buffer before homogenization. The homogenate was then centrifuged for 10 min at 2000×*g* (4°C) to remove fat. The resulting intermediate phase was centrifuged for 45 min at 32000 rpm using a Beckman Ultracentrifuge XL-70. The pellet was re-suspended in 100 μl of Lysis Buffer (80 mmol/L NaCl, 50 mmol/L Tris-HCl, 2 mmol/L CaCl_2_, 1% TritonX-100 and protease inhibitor cocktail), and immediately frozen at –80°C. After protein determination (DC™ protein assay, Bio-Rad Laboratories, Hercules, USA) membranes were pooled group-wise. Reduced pooled membranes (10, 20, 30 μg or 40, 60, 80 μg protein) were separated on a NuPage 3–8% Tris-Acetate gel and then transferred onto nitrocellulose membranes (Invitrogen, Carlsbad, USA). After blocking in 5% non-fat dry milk in PBS-T (PBS with 0.1% Tween-20), the nitrocellulose membranes were incubated overnight at 4°C with the primary antibody (Ab) specific for the protein of interest in 5% non-fat milk powder in PBS-T. ABCA1 was detected with mouse monoclonal Ab (1∶1000, ab18180, Abcam LtD, Cambridge, UK) and as secondary antibody a peroxidase-conjugated goat anti-mouse antibody was used (1∶20000; Pierce Biotechnology, Inc., Rockford). SR-BI was detected with rabbit polyclonal Ab (1∶3000, ab396, Abcam LtD, Cambridge, UK) and a peroxidase-conjugated donkey anti-rabbit antibody (1∶60000; GE Healthcare, UK). LDLr protein was detected with rabbit monoclonal Ab (1∶3000, ab52818, Abcam LtD, Cambridge, UK) and a peroxidase-conjugated donkey anti-rabbit antibody (1∶60000; GE Healthcare, UK). The specific bands were detected using SuperSignal chemiluminescence kit (Pierce Biotechnology, Inc., Rockford) and Bio-Rad Universal Hood II and quantified by Bio-Rad Quantity One software (Bio-Rad Laboratories, Hercules, USA). Signals were plotted by μg-loaded protein and the slope of the curves was calculated by method of least square. The slope of the ASOctrl group was set equal to 100%.

### Serum and liver lipid analysis

Serum lipoproteins were fractionated by size from 12 μL of individual serum samples using a Superose 6 PC 3.2/30 column (GE Healthcare Bio-Sciences AB, Uppsala, Sweden) as previously described [18]. The respective lipoprotein fraction lipid concentrations were calculated after integration of the individual chromatograms. ApoAI and apoE content was determined in the lipoprotein fractions by western blot analysis using an anti-mouse apoA-I (Rockland, Gilbertsville, PA, USA) and an anti-mouse apoE (Calbiochem, Merk, Darmstadt, Germany) as primary antibody. Lipids were extracted from ∼100 mg liver sample using 6 mL chloroform-methanol (2∶1 v/v). The organ was removed from the tubes containing the lipid extract, which was then dried down under N_2_ and re-dissolved in a measured volume of 2∶1 chloroform/methanol. Diluted H_2_SO_4_ was added to the extract, which was then vortexed and centrifuged to split the phases. The aqueous upper phase was aspirated and discarded, and an aliquot of the bottom phase was removed and dried down. 1% Triton X-100 in chloroform was then added, and the solvent was evaporated [19]. Lipids were quantified on Tecan GENios plate reader equipped with Magellan Software (Tecan Group Ltd, Switzerland) using the respective enzymatic kit: Cholesterol/HP, Triglyceride/GB (Roche Diagnostics, Indianapolis, USA), and Free Cholesterol C (Wako Chemicals USA Inc.). The amount of esterified cholesterol was calculated by subtracting the unesterified cholesterol from the total cholesterol, and this difference was multiplied by 1.67 to convert it to CE mass. Liver lipid levels were normalized for the hepatic protein content measured according to Lowry method in the tissues digested with NaOH (1 mol/L). To obtain a relative index of hepatic bile acid synthesis, the concentration of 7α-hydroxy-4-cholesten-3-one (C4) was assayed in the liver lipid extract by isotope dilution-mass spectrometry as previously reported, and the ratio of C4 to total cholesterol was calculated [20], [21].

### Cell culture and materials

Cell culture media, trypsin-EDTA, and gentamicin were purchased from Gibco/Invitrogen (Paisley, Scotland). Fetal bovine serum (FBS), serum albumin, the ACAT inhibitor Sandoz 58-035, and 8-(4-Chlorophenylthio)adenosine 3′,5′-cyclic monophosphate sodium salt (cpt-cAMP) were from Sigma-Aldrich (Stockholm, Sweden). [1,2-^3^H(N)]-cholesterol was from PerkinElmer (Uppland Väsby, Sweden). Tissue culture flasks, plates, and tubes were from Thermo Fisher Scientific/Nunc (Roskilde Site, Denmark) or Falcon (Lincoln, NY, USA). Human serum, prepared from blood of healthy donors, was provided by the Dept. of Clinical Immunology and Transfusion Medicine, Karolinska University Hospital, Huddinge, Sweden (http://www.karolinska.se/Karolinska-Universitetslaboratoriet/Kliniker/Immunologi-transfusionsmedicin/). The blood was donated for general research purposes. The donors gave informed consent that the blood could be used for preparation of serum for research purposes, but were not explicitly asked about consent to publish data. All samples were anonymized, i.e. not possible to trace back to the blood donor. Ethical permission was not warranted for requiring blood components per se, as this did not pose any extra harm to the donors (regular blood donation was performed). Lipid poor apoA-I isolated was isolated and purified from human serum as previously described [22]. J774.A1 murine macrophages were purchased from American type culture cell (LGC standards AB), and maintained in RPMI with L-Glutamine plus 10% FBS and gentamicin in 5% CO_2._ Fu5AH rat hepatoma cells [23]–[26] were a kind gift of Prof. Franco Bernini (Dept. of Pharmacy, University of Parma, Italy). Fu5AH were cultured in High glucose DMEM plus 10% FBS and gentamicin. Ultima Gold™^ was from PerkinElmer (Uppland Väsby, Sweden).^

### Quantification of serum cholesterol efflux capacity (CEC)

Mouse sera from the 4-weeks and 0-weeks experiment were tested as cholesterol acceptors in different cell models to evaluate the CEC. J774 murine macrophages incubated with cpt-cAMP were used as a model of total cholesterol efflux, since all the efflux pathways are active [27], [28]. J774 cultured under basal conditions were used to evaluate the aqueous diffusion [26], [27]. The ABCA1-mediated cholesterol efflux was the difference between the cholesterol efflux measured by J774 incubated with cpt-cAMP and the cholesterol efflux measured by J774 cultured in basal condition [26], [27]. SR-BI mediated cholesterol efflux was measured using Fu5AH rat hepatoma cells, a stable highly SR-BI-expressing cell line [29]. In brief, cells were plated into 24-well plates in medium containing 10% FBS. Monolayers were washed with PBS and incubated for 24 h in medium containing 1% FBS, [1,2-^3^H(N)]-cholesterol and ACAT inhibitor (2 μCi/mL). Cells were then incubated for 18 h with medium plus 0.2% BSA and ACAT inhibitor (2 μCi/mL), with 0.3 mmol/L cpt-cAMP when appropriate. Cells were then harvested with NaOH (1 M) and counted by liquid scintillation. These cells provided baseline (time 0) values for total [1,2-^3^H(N)]-cholesterol content. Cell monolayers were then incubated with 1% (v/v) mouse serum in medium for 4 h. Cell media were centrifuged to remove floating cells, and radioactivity in the supernatant was determined by liquid scintillation counting. Cholesterol efflux was calculated as: (cpm in medium at 4 h/cpm at time 0) x 100. For each experiment aimed to measure the serum CEC by ABCA1, we monitored ABCA1 up-regulation in J774 cells as increased efflux to human apoA-I (20 μg/mL) from cells treated with cpt-cAMP compared to untreated cells. Pooled human serum from 20 healthy donors was tested in every experiment as an acceptor (2% v/v) in order to monitor and correct for the inter-assay variability in cholesterol efflux.

### 2D gel electrophoresis

Serum HDL subclasses were separated by 2D electrophoresis, in which agarose gel electrophoresis was followed by non-denaturing polyacrylamide gradient gel electrophoresis and subsequent immunoblotting [23]. In the first dimension, serum (5 μL) was run on a 0.5% agarose gel; agarose gel strips containing the separated lipoproteins were then transferred to a 3-20% polyacrylamide gradient gel. Separation in the second dimension was performed at 30 mA for 4 h. Fractionated HDLs were then electroblotted onto a nitrocellulose membrane and detected with an anti-mouse apoA-I (Rockland, Gilbertsville, PA, USA) or an anti-mouse apoE (Calbiochem, Merck, Darmstadt, Germany) antibody and visualized by enhanced chemiluminescence (GE Healthcare Biosciences, Uppsala, Sweden). Densitometric analysis was performed with a GS-690 Imaging Densitometer and the Multi-Analyst software (Bio-Rad Laboratories, Hercules, CA, USA). Serum content of preβ-HDL was expressed as percentage of total apoA-I.

### RNA extraction, cDNA synthesis, and qPCR analysis of mRNA expression levels

RNA from liver tissue was isolated using RNeasy Mini Kit (QIAGEN GmbH, Hilden, Germany) according to the manufacturer's protocol. The concentration and quality of the purified total RNA were determined spectrophotometrically at OD_260_ nm and by the OD_260/280_ ratio, respectively. Synthesis of single-stranded cDNA was carried out on 0.5 μg RNA using iScript cDNA synthesis kit (Bio-Rad Laboratories, Inc., Hercules, CA) following a standard protocol. PCR primers were designed using Primer Express Software version 2.0, a program especially provided for primer design using ABI qPCR machines. qPCR assay on the basis of SYBR Green I technology was performed with ABI 7500 fast qPCR system (Applied Biosystems, Foster City, CA). For each pair of primers, a dissociation curve analysis was conducted to validate the specificity of the PCR amplification. Primers were used at a concentration of 100 mmol/L in qPCR analyses and the sequences are listed in Table S1. We calculated relative changes employing the comparative method using *Tfiib* as the reference gene and controls as calibrators as indicated in the figures.

### Statistical analysis

Statistics was calculated using Statistica software (Stat Soft Inc., USA). As indicated in the text or in each figure, differences between the treatment groups were determined by the Mann Whitney test. Multi-way analysis of variance (ANOVA) test followed by post-hoc comparisons of group means according to the least significant difference (LSD) method was used when comparing the effect of treatment in the different mouse genotypes. Outlier rejection was performed prior to the analysis. Mean, standard error of the mean (SEM), and p-values were used for descriptive purposes. A p-value <0.05 was considered statistically significant.

## Results

**Figure 1:**
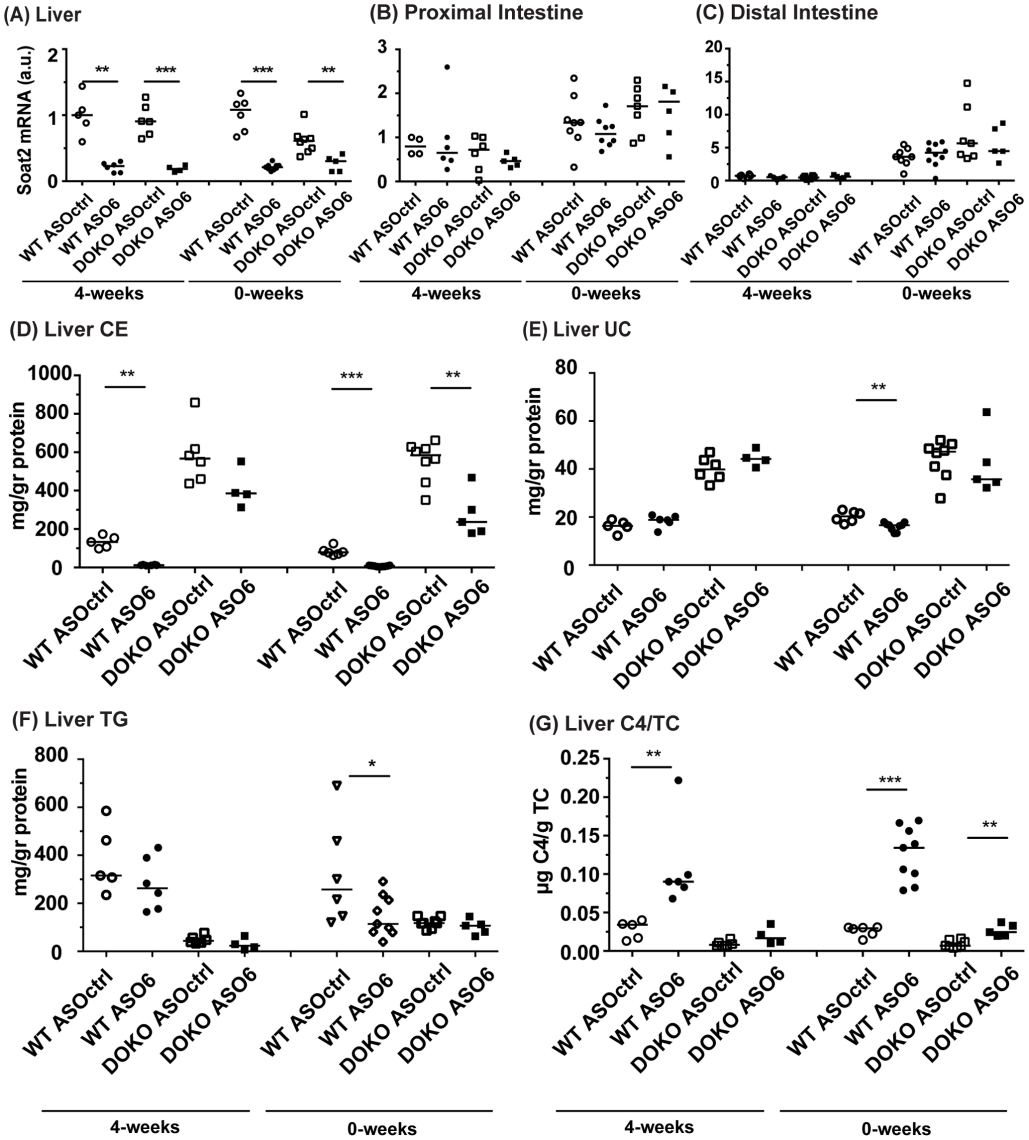
Effect of hepatic ACAT2 down regulation on *Soat2* expression, and on the liver lipid content. *Soat2* mRNA was quantified by real-time RT-PCR in samples from liver (A), proximal (B) and distal intestine (C). Data were standardized for *Tfiib* mRNA expression, and normalized to WT Ctrl in each experiment. Cholesteryl ester (CE; D), unesterified cholesterol (UC; E), and triglyceride (TG; F) mass was measured in liver lipid extracts by enzymatic assays as described in the methods section. Cholesteryl esters were calculated subtracting the mass of unesterified cholesterol to total cholesterol, and adjusted for the mass of the moiety of fatty acid in CE. Liver lipid levels were normalized for the hepatic protein content. 7α-hydroxy-4-cholesten-3-one (C4; G) mass was measured in the liver lipid extracts by LC-MS/MS, and normalized for the hepatic total cholesterol (TC) mass. Error bars represent the median. Mann Whitney test, * p<0.05, ** p< 0.01, *** p<0.001.

In order to control for the efficacy of ASO6 treatment, we examined *Soat2* expression in the two organs where ACAT2 is exclusively expressed [3], the intestine and liver. In agreement with previous studies [13], ASO6 administration led to a specific down-regulation of ACAT2 in the liver (Figure 1 A) without modifying the expression in proximal or distal intestine (Figure 1 B, C). When the feeding with the western type of diet was started 4 weeks after the ASO injection (4-weeks), ASO6 reduced hepatic *Soat2* mRNA by about 80% in both WT and DOKO mice. The reduction of *Soat2* was coupled with more than 80% reduction in the liver microsomal activity of ACAT2 in both genotypes (ASO6 vs ASOctrl: 0.08±0.01 vs 1.21±0.14 ng/mg/min in WT, p<0.005; and 0.15±0.03 vs 0.86±0.06 ng/mg/min, p<0.01; in DOKO respectively). Also when the diet challenge was started concomitantly with the treatment (0-weeks), a similar reduction of liver microsomal activity of ACAT2 was observed in both WT (ASO6 vs ASOctrl: 0.03±0.004 vs 0.55±0.10 ng/mg/min; p<0.005) and DOKO (ASO6 vs ASOctrl: 0.14±0.02 vs 0.68±0.06; p<0.005) mice. As seen in the 4-weeks experiment, the reduction in ACAT2 activity followed a significant reduction in hepatic *Soat2* mRNA expression in both genotypes (Figure 1A).

### Liver lipid content in ASO6 treated mice

We evaluated the lipid content in the liver samples from all the experiments performed. As ASO6 treatment reduced hepatic ACAT2 activity more then 80% in both the 4-weeks and 0-weeks experiment, hepatic CE mass was diminished. As shown in Figure 1D, ASO6 treatment in the 4-weeks experiment reduced hepatic CE levels by 91% in WT mice, whereas only a 30% reduction was observed in DOKO mice receiving ASO6. A similar effect was observed in the 0-weeks experiment where ASO6 lowered hepatic CE levels up to 91% in WT and up to 50% in DOKO mice. Hepatic ACAT2 knock down by ASO6 did not result in accumulation of UC in the liver as previously observed in a different mouse genotype [13]. In the 4-weeks experiment hepatic UC was not affected by ASO treatment in either WT or DOKO mice, whereas in the 0-weeks experiment ASO6 led to a 21% reduction of hepatic UC only in WT mice (Figure 1E). ACAT2 knockdown has been reported to alter triglyceride (TG) metabolism in mice, thus lowering hepatic TG levels [30]. We quantified the hepatic TG content in both the 4-weeks and 0-weeks experiments (Figure 1F). In WT mice ASO6 lowered hepatic TG, although significantly only in the 0-weeks experiment. Liver TG mass was not affected by ASO6 in DOKO mice, which showed decreased hepatic TG levels compared to their wild type counterparts independently of the treatment (p<0.001 and p<0.01 in the 4-weeks and 0-weeks experiments respectively; Factorial ANOVA). In order to assess whether hepatic ACAT2 knock down induces BA synthesis, and to confirm that LXR DOKO mice have a reduced synthesis, we also quantified hepatic C4. As shown in Figure 1G, in both experimental conditions ASO6 led to an increase of liver C4 content in both WT and DOKO mice. As expected C4 levels in DOKO mice were significantly lower that in WT mice independently of the treatment (4-weeks and 0-weeks: p<0.001, Factorial ANOVA).

### ACAT2 activity and ABCA1 expression in the liver

**Figure 2:**
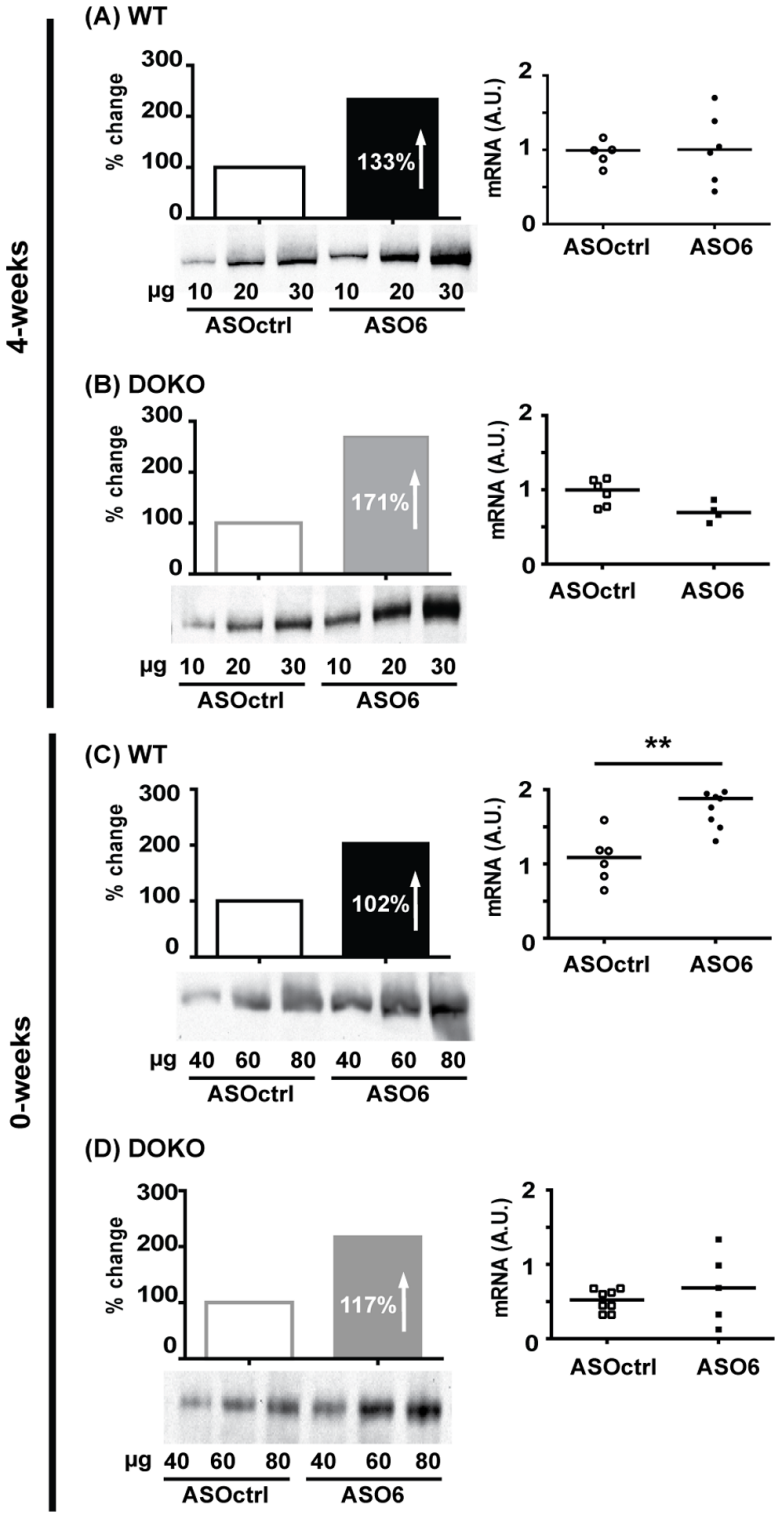
Hepatic ACAT2 down regulation increases the ABCA1 protein independently of the mRNA expression. Liver membrane proteins were pooled group-wise, loaded, and separated on Tris-Acetate Gels. After transfer onto nitrocellulose membrane, samples were incubated with anti-mouse ABCA1 antibody. ABCA1 band (≈ 250 kD) was detected by chemiluminescence, and signals were plotted by μg-loaded protein. The slope of the curves was calculated by method of least square, and the slope of the ASOctrl group was set equal to 100%. Hepatic *Abca1* mRNA was quantified by real-time RT-PCR. Data were standardized for *Tfiib* mRNA expression, and normalized to WT Ctrl in each experiment. In the mRNA data error bars represent the median. Mann Whitney test, ** p< 0.01.

Since we did not observe any accumulation of UC following hepatic ACAT2 down-regulation, we hypothesized that UC could be channelled into nascent HDL through the ABCA1 transporter, whose expression was analysed at both mRNA, and protein levels in liver membranes. In the 4-weeks experiment, ASO6 treatment of WT induced a 133% increase in hepatic ABCA1 protein, whereas no effects were observed at the mRNA level (Figure 2A). In DOKO mice (Figure 2B) ASO6 led to a 171% increase of the ABCA1 protein compared to ASOctrl. *Abca1* mRNA was slightly reduced by ACAT2 down-regulation in this mouse genotype. Also in the 0-weeks experiment, hepatic ACAT2 depletion by ASO6 led to about a doubling of the ABCA1 protein expression in liver membranes of both WT and DOKO mice (Figure 2 C and D. However, under these experimental conditions the *Abca1* mRNA levels increased in WT mice (Figure 2C).

### Serum lipid lipoprotein profile

**Figure 3:**
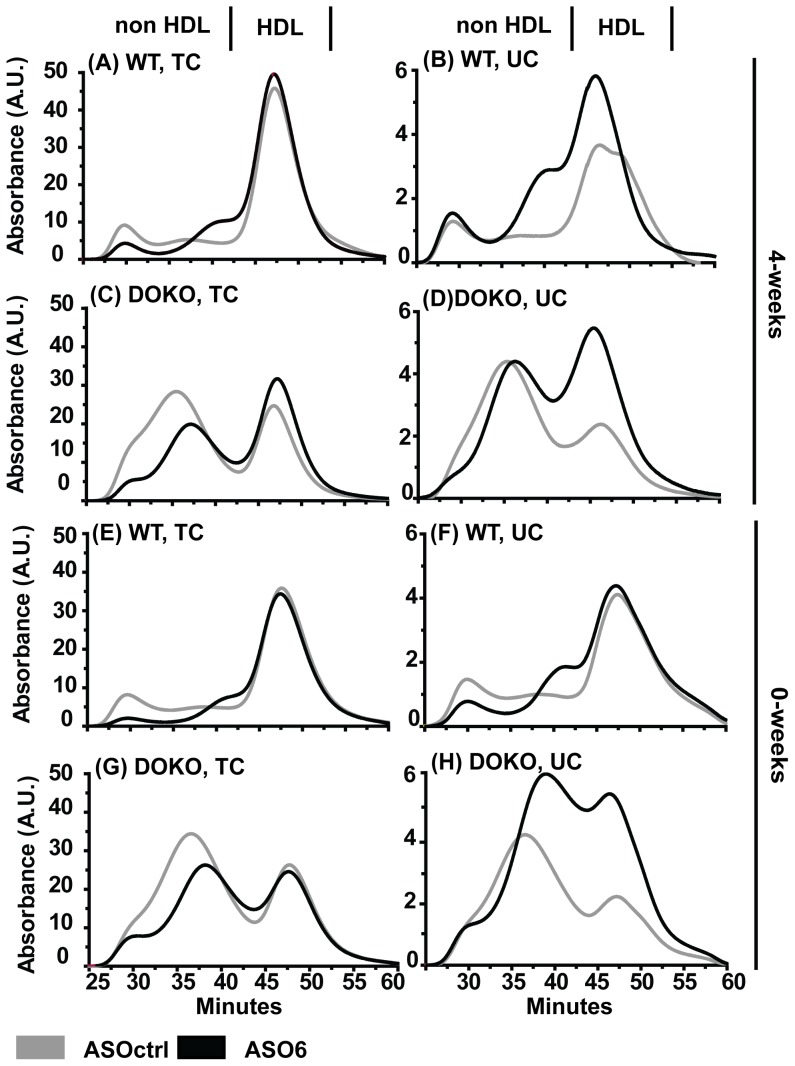
Effect of hepatic ACAT2 down regulation on serum lipoprotein profile. Serum lipoproteins were separated by size exclusion chromatography, and the total (left panels) and unesterified cholesterol (right panels) content was determined by a system for on-line detection. Black solid lines are the average chromatogram for the ASOctrl treated group, and grey solid lines are the average chromatogram for the ASO6 treated group (n = 6–8). Lipoprotein profiles of WT mice are shown in panels A, B, E, and F. Lipoprotein profiles of LXR DOKO mice are represented in panels C, D, H and G.

**Figure 4:**
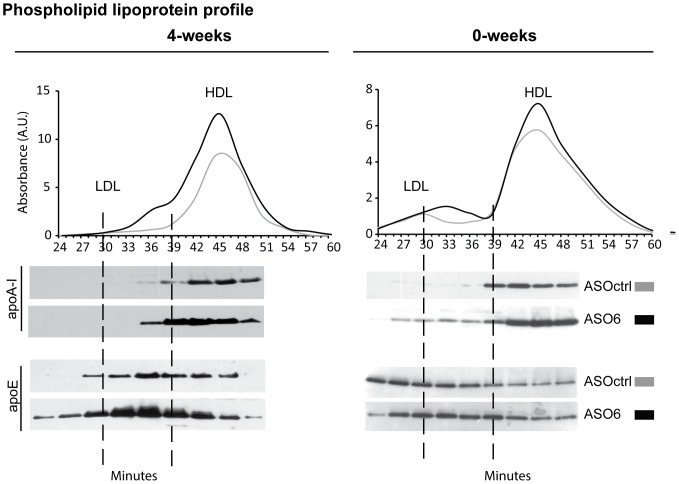
Hepatic ACAT2 down regulation led to formation of apoE-rich HDL. Serum samples from WT mice treated with ASO6 or ASOctrl were pooled (n = 3–6) and ultracentrifugated. Lipoproteins with a density < 1.21 g/L were then separated by SEC, collecting the lipoprotein fractions every 3 min. As described in the method section, apolipoprotein (apo) AI and E content (A) were determined in the different fractions by western blot analysis. Phospholipids (PL) were quantified enzymatically.

The analysis of the serum lipid profile revealed that in WT mice ASO6 did not affect the total levels of TC or UC (Table 1). Instead it led to a redistribution of cholesterol within the different lipoprotein fractions (Figure 3 A, B, E, F). As expected the reduction of ACAT2 expression in the liver reduced TC in the VLDL fractions (4-weeks: 0.25±0.02 vs 0.56±0.09 mmol/L, p<0.005; 0-weeks: 0.15±0.03 vs 0.66±0.09 mmol/L, p<0.005). Interestingly in the TC serum lipoprotein profile, ASO6 treatment led to the appearance of a new peak between LDL and HDL particles in both experiments (Figure 3A and E). This effect was even more pronounced when the distribution of UC in serum lipoproteins was analysed (Figure 3B, F), and it was also present when phospholipids (PL) were analysed (Figure 4). In order to further characterize this new peak, pooled serum from WT mice from the 0-weeks experiment was separated by size exclusion chromatography (SEC), and lipoprotein fractions were collected. Analysis of apoA-I and apoE content by Western blot revealed an increased content of both these apolipoproteins in the fractions (nr 30-39) corresponding to the lipoprotein peak formed by ASO6 treatment (Figure 4). In DOKO mice ASO6 treatment did not change the plasma levels of TC, but led to an increase in serum UC (Table 1). This effect was more pronounced in the 0-weeks experiment. In the 4-weeks experiment DOKO mice receiving ASO6 showed higher levels of TC in the HDL lipoprotein fractions compared to ASOctrl treated animals (Figure 3E, F). In both experiments ASO6 treatment strongly increased the HDL-UC in DOKO mice (Table 1 and Figure 3D, H). In both WT and DOKO mice from the 4-weeks experiment down-regulation of ACAT2 increased serum TG levels in the apoB-containing lipoprotein fraction (1.16±0.06 vs 0.74±0.09 mmol/L in WT, p<0.01; 0.29±0.05 vs 0.09±0.01 mmol/L in DOKO, p<0.01). In the 0-weeks experiment, this effect was only observed in DOKO mice (0.72±0.08 vs 0.52±0.05 mmol/L in WT; 0.35±0.03 vs 0.08±0.01 mmol/L in DOKO, p<0.005).

**Table 1:** Lipid quantification in serum and HDL lipoproteins.

	4-weeks				0-weeks			
mmol/L	WT ASOctrl	WT ASO6	DOKO ASOctrl	DOKO ASO6	WT ASOctrl	WT ASO6	DOKO ASOctrl	DOKO ASO6
**TC**	5.30±0.33	5.54±0.21	6.10±0.30	5.30±0.27	4.87±0.36	4.26±0.41	7.67±0.24	6.33±0.59
**HDL-TC**	4.23±0.26	4.62±0.17	2.15±0.05	2.87±0.28	3.78±0.34	3.68±0.33	2.65±0.12	2.49±0.39
**UC**	1.45±0.11	1.64±0.17	1.45±0.07	2.01±0.29	1.29±0.11	1.39±0.14	2.55±0.14	4.66±0.88*
**HDL-UC**	1.13±0.12	1.17±0.14	0.41±0.01	1.04±0.10†	0.91±0.08	1.04±0.10	0.72±0.06	1.94±0.27†
**PL**	5.87±0.61	7.07±0.29	4.45±0.23	6.41±0.60*	5.65±0.26	5.33±0.27	5.11±0.21	5.44±0.57
**HDL-PL**	5.03±0.61	6.45±0.32	2.46±0.17	4.60±0.17†	4.82±0.22	4.61±0.22	2.50±0.37	3.54±0.39
**TG**	1.09±0.13	1.29±0.06	0.22±0.02	0.43±0.05*	0.58±0.04	0.78±0.07	0.13±0.01	0.48±0.11‡
**HDL-TG**	0.35±0.09	0.13±0.03	0.12±0.03	0.14±0.01	0.06±0.01	0.06±0.01	0.06±0.01	0.05±0.02

Serum lipoproteins were separated by size exclusion chromatography, and the concentration of total (TC) and unesterified (UC) cholesterol, phospholipids (PL), and triglycerides (TG) was determined by a system for on-line detection. Data are expressed as average ± SEM (n = 6–8). Mann Whitney test, *p<0.05, † p<0.01, ‡ p<0.005.

### Effect of ACAT2 disruption on serum cholesterol efflux capacity (CEC)

**Figure 5:**
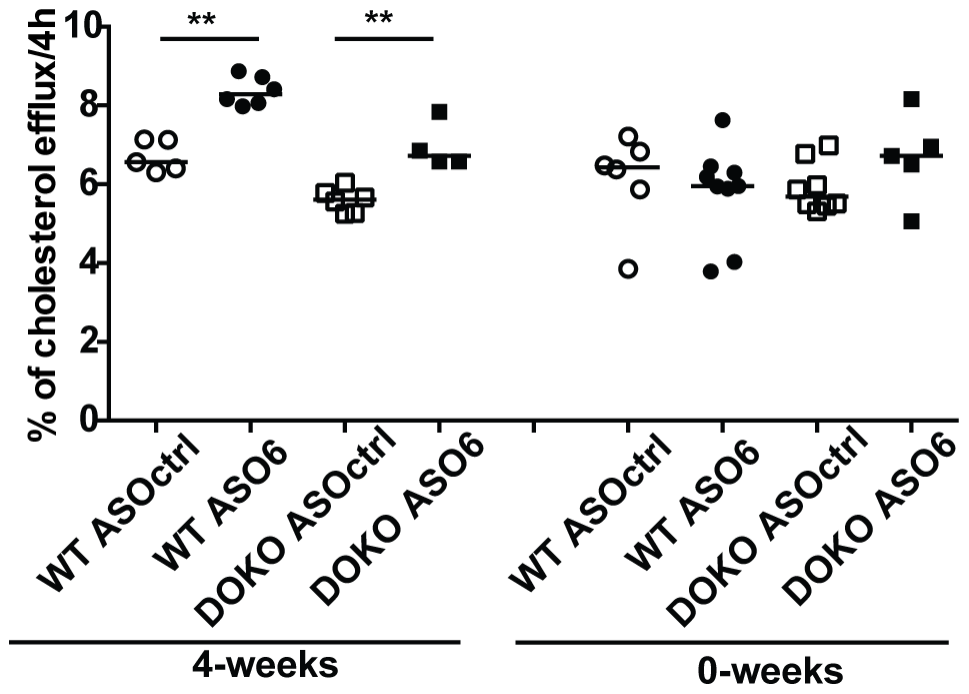
Impact of hepatic ACAT2 down regulation on serum CEC. J774 murine macrophages were radiolabeled with [^3^H]-cholesterol for 24 h, equilibrated in a BSA-containing medium ± cpt-cAMP 0.3 mmol/L for 18 h. Efflux was measured after 4 h of cell incubation with medium containing 1% serum from WT (black bars) and LXR DOKO (grey bars) mice treated with ASOctrl (empty bars) or ASO6 (filled bars). Efflux is expressed as cpm in medium/cpm T0 × 100. Error bars represent the median. Mann Whitney test, ** p<0.01.

Since changes in serum lipid profiles were observed in both WT and DOKO mice treated with ASO6, we investigated whether the effect on HDL fractions would affect the capacity of serum to accept cholesterol from macrophages. CEC is strictly related to the composition of the lipoproteins present in the serum, and more importantly to the capacity of the different HDL subclasses to act as a lipid acceptor [26]. Thus, serum samples from all the experiments were tested using cAMP-treated J774 macrophages as cholesterol donors [27]. As shown in Figure 5, serum from both WT and DOKO mice treated with ASO6 was a more efficient cholesterol acceptor than serum from ASOctrl treated mice, but this was seen only in the 4-weeks experiment. No differences in serum CEC were observed comparing serum from ASO6 treated WT and DOKO mice with their respective control from the 0-weeks experiment.

### Effect of ASO6 on HDL subclasses

**Figure 6:**
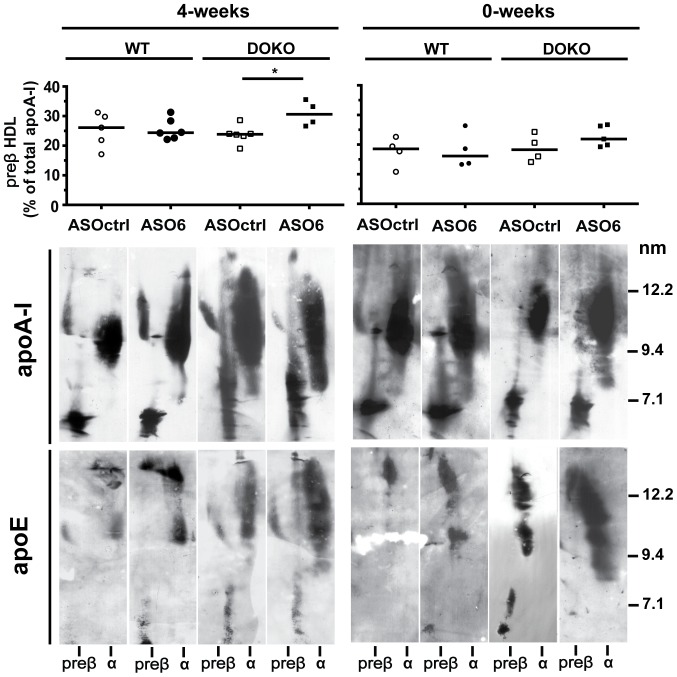
Hepatic ACAT2 knock-down modifies HDL subclasses. HDL subclasses were separated by non-denaturing two-dimensional gel electrophoresis and transferred onto a nitrocellulose membrane, on which lipoproteins were detected with an anti-mouse apoA-I or apoE antibody; a representative animal in each group is shown. Preβ-HDL content was calculated as percentage of total apoA-I signal from 2D-electrophoresis developed against apoA-I. Error bars represent the median. Mann Whitney test, * p<0.05.

The presence of specific HDL subclasses determines HDL functionality and serum CEC. Therefore, we investigated whether ASO6 could affect the distribution of the HDL particle, providing us with a possible explanation for the effects we observed on the lipid lipoprotein profile and serum CEC. HDL were separated by 2D gel electrophoresis, and immunoblotted against apoA-I or apoE. As shown in Figure 6 (left panels), in WT mice from the 4-weeks experiment ASO6 treatment did not affect the pre-β-HDL, but led to the appearance of larger α-particles. Conversely in DOKO mice, hepatic ACAT2 down-regulation induced the appearance of smaller α-HDL, and significantly increased the pre-β-HDL content. For both mouse genotypes from the 0-weeks experiment, no differences were observed in HDL subclasses between ASO6 and ASOctrl treatment (Figure 6 right panels).

In both experiments ASO6 increased the amount of apoE-containing HDL, without modifying the particle size (Figure 6 bottom panels). In the 4-weeks experiment we observed an average of 26% increase in apoE-containing HDL when WT ASO6 treated mice were compared to randomly selected ASOctrl treated animals. For DOKO mice the average increase was 128%. In the 0-weeks experiment the average increase in apoE-containing HDL of ASO6 treated WT mice was of 122%, while in DOKO mice was about 100%. In both genotypes from the 4-weeks and 0-weeks experiment, apoE-containing HDL from ASO6 treated animals showed a progressive shift of migration towards preβ position.

### Changes in HDL subclasses and cholesterol efflux mechanisms

In the 4-weeks experiment, hepatic ACAT2 down-regulation led to changes in apoA-I-containing particle distribution and resulted in increased capacity of serum to promote cholesterol efflux from macrophages. Since cholesterol efflux occurs by several mechanisms characterized by a preferential acceptor [31], serum samples from the 4-weeks experiment were tested for their cholesterol acceptor capacity using different cell lines selectively expressing specific lipid transporters. Reflecting the presence of larger mature HDL secondary to hepatic ACAT2 knockdown, serum from ASO6 treated WT mice showed increased capacity to promote cholesterol efflux by aqueous diffusion and by SR-BI transporter when compared to serum from ASOctrl treated WT (Figure S1 A and B). Serum from ASO6 treated DOKO mice was more efficient than serum from DOKO mice treated by ASOctrl, in promoting cholesterol efflux through the ABCA1 transporters (Figure S1 C). Indeed, in DOKO mice ASO6 treatment resulted in increased serum levels of pre-β-HDL (Figure 4), which have been demonstrated to be a preferential acceptor for the ABCA1-mediated efflux [32]. Efflux via aqueous diffusion (AD) was also increased to serum from DOKO mice when hepatic ACAT2 was reduced.

### Effect of hepatic ACAT2 down-regulation on genes involved in lipid metabolism

The reduced hepatic ACAT2 activity by ASO treatment ought to affect the pool of UC that can signal to and activate the LXR system. Surprisingly, no consistent LXR activation was observed in any of the experiments, as assessed by the hepatic gene profile of LXR target genes (Table 2). *Cyp7a1* and *Abcg5*; were not affected by the ASO6 treatment, whereas *Abcg8* and *Scd1* were respectively increased only in WT and DOKO from the 0-weeks experiment. A decreased hepatic ACAT2 activity appeared to result in a down-regulation of the genes under control of the SREBP2 system, but only in WT mice from the 4-weeks experiment (Table 2). Indeed ASO6 treatment resulted in reduced mRNA expression of *Ldlr, Pcsk9*, *Hmgcr* and *Hmgcs* (see Table 2 and Figure S2). Some of these effects were also seen in DOKO mice (Table 2). However, hepatic ACAT2 down-regulation did not reduce the expression of SREBP2 target genes when the dietary challenge was started together with ASO6 treatment (0-weeks; Table 2). As shown in Figure S2, LDLr protein expression was analysed in liver membrane pools. In the 4-weeks experiment hepatic ACAT2 down-regulation did not affect LDLr protein in WT (Figure S2 A), whereas it reduced the receptor by 98% in DOKO mice (Figure S2 C). A similar reduction was also observed in DOKO animals from the 0-weeks experiment (Figure S2 G).

**Table 2:** Hepatic expression of genes involved in lipid metabolism.

	4-weeks				0-weeks			
Gene (a.u.)	WT ASOctrl	WT ASO6	DOKO ASOctrl	DOKO ASO6	WT ASOctrl	WT ASO6	DOKO ASOctrl	DOKO ASO6
***Srebp2***	0.92±0.04	0.63±0.13	1.25±0.17	0.93±0.05	0.99±0.14	1.17±0.41	0.30±0.04	0.30±0.01
***Hmgcr***	1.15±0.12	0.524±0.10*	0.84±0.06	0.57±0.05*	2.77±1.03	1.88±0.33	0.17±0.02	0.28±0.10
***Hmgcs***	1.25±0.23	0.43±0.16*	0.90±0.09	0.55±0.11*	0.40±0.13	0.32±0.08	0.10±0.01	0.18±0.06*
***Pcsk9***	1.51±0.39	0.18±0.07†	0.23±0.08	0.08±0.02	1.47±0.35	1.67±0.48	0.19±0.05	0.30±0.14
***Lcat***	0.75±0.09	0.81±0.15	0.96±0.10	0.65±0.11	0.78±0.07	0.81±0.04	0.23±0.03	0.25±0.0
***Lipc***	1.32±0.21	1.50±0.29	0.81±0.09	0.67±0.24	0.71±0.21	0.68±0.11	0.23±0.0	0.15±0.07
***Cyp7a1***	2.30±1.17	4.23±1.10	1.29±0.25	0.80±0.16	1.74±0.50	1.98±0.37	0.24±0.08	0.37±0.16
***Abcg5***	1.37±0.22	1.18±0.30	0.24±0.03	0.16±0.02	2.78±1.03	1.88±0.33	0.17±0.02	0.28±0.10
***Abcg8***	1.54±0.26	1.74±0.60	0.34±0.03	0.24±0.04	1.54±0.23	2.63±0.41*	0.65±0.10	0.45±0.12
***Scd1***	2.86±0.77	1.69±0.52	0.02±0.01	0.02±0.00	1.43±0.27	1.14±0.17	0.004±0.00	0.01±0.01*
***Fasn***	1.50±0.33	0.15±0.05‡	0.04±0.01	0.03±0.01	1.45±0.56	1.47±0.31	0.05±0.00	0.05±0.02
***Acc1***	1.50±0.33	0.42±0.10‡	0.38±0.02	0.26±0.03	1.43±0.27	1.13±0.17	0.004±0.00	0.01±0.01*
***Srebp1c***	0.60±0.15	0.34±0.17	0.001±0.0	0.00±0.00	0.61±0.14	1.21±0.23	0.00±0.00	0.00±0.00
***Tgh1***	1.84±0.30	1.64±0.17	0.50±0.04	0.72±0.0†	1.29±0.18	1.47±0.36	0.26±0.04	0.37±0.15
***Tgh2***	1.55±0.16	1.44±0.04	0.17±0.03	0.07±0.01	1.26±0.20	0.84±0.21	0.05±0.01	0.13±0.07
***Ctsd***	3.94±1.88	0.68±0.17	5.78±1.45	2.87±0.47	0.83±0.14	0.91±0.20	1.51±0.24	0.77±0.24
***Zdhhc8***	1.13±0.23	1.28±0.24	3.02±0.96	2.40±0.53	0.59±0.09	0.74±0.12	0.49±0.06	0.47±0.10
***Rab8a***	0.84±0.07	1.06±0.24	2.26±0.41	1.59±0.39	0.66±0.10	0.94±0.20	0.28±0.02	0.43±0.14

mRNA was quantified by real-time RT-PCR. Data were standardized for *Tfiib* mRNA expression, and normalized to the expression of each gene in WT Ctrl in each single experiment. mRNA data are expressed as average ± SEM (n = 6–8). Mann Whitney test, *p<0.05, † p<0.01, ‡ p<0.005.

In spite of the minor effects seen for TG content in the liver of WT animals (see above), in the 4-weeks experiment ASO6 treatment reduced the mRNA of genes involved in fatty acid synthesis, which was likely mediated by a SREBP1-c down-regulation (i.e. *Fasn* and *Acc1*; Table 2). However, in the 0-weeks experiment, we did not observe this effect. In these experimental conditions, ASO6 increased *Scd1* and *Acc1* mRNA levels in the liver of DOKO mice (Table 2). Triacylglycerol hydrolase 1 and 2 mRNA expression was significantly reduced in LXR DOKO mice compared to WT independently of the treatment (4-weeks: *Tgh1*, p<0.01 and *Tgh2,* p<0.001; 0-weeks: *Tgh1*, p<0.001 and *Tgh2,* p<0.001; Factorial ANOVA). ASO6 did affect the expression of these genes in WT mice (Table 2). Only in the 4-weeks experiment and in DOKO mice did ACAT2 down-regulation lead to slightly increased *Tgh1* mRNA levels (Table 2). We also evaluated the hepatic expression of the HDL receptor SR-BI, both at mRNA and protein levels. As shown in Figure S2 (right panels), hepatic ACAT2 down-regulation led to a reduction of SR-BI protein expression in the liver membranes, without affecting the mRNA. In the 4-weeks experiment ASO6 reduced SR-BI by 28% and 6% in WT and DOKO respectively (Figure S2 B and D). In the 0-weeks experiment hepatic ACAT2 down-regulation led to a reduction in SR-BI protein expression by 8% in WT and 36% in DOKO mice (Figure S2 F and H).

## Discussion

Our main aim with this study was to uncover a possible link between hepatic ACAT2 activity and HDL metabolism in mice. We hypothesized that the excess of UC in the hepatocytes due to a lower activity of ACAT2 may be also shunted into nascent HDL by ABCA1. In order to better unravel this mechanism we decided to include LXR α/β double knockout mice in our experiments. In these animals UC should be more available to secretion in to nascent HDL because of lower bile acid synthesis paralleled by a maintained ABCA1 expression in the liver.

We have previously reported that the hepatic ABCA1 protein was up-regulated when the expression of hepatic ACAT2 was reduced, independently of the levels of *Abca1* mRNA expression [33]; an effect also seen in apoB100only-LDLr^−/−^ mice [13]. In mice where ACAT2 was selectively knocked out in the liver, hepatic *Abca1* mRNA was increased [14]. However, the protein expression of ABCA1 was not reported in these studies. Here we clearly demonstrate that down-regulation of hepatic ACAT2 activity results in a strong stimulation of ABCA1 protein expression in liver membranes, and that this process is definitively independent from LXRs. The up-regulation of the ABCA1 protein expression results from an as yet unidentified post-transcriptional process, since it is clearly independent of *Abca1* mRNA expression. Intracellular UC may play a role since it has been shown that UC loading induces ABCA1 expression in several cell models [34]. Wang et al showed that hepatocytes from mice depleted of NPC1 accumulate FC and have an up-regulation of ABCA1 protein. Cathespin D, a lysomal proteinase, has been proposed as a possible player in this upregulation [35]. Furthermore Rab8 over-expression was found to increase ABCA1 protein in human primary macrophages, without affecting the mRNA levels [36]. Finally, palmitoylation of ABCA1 by the palmitoyl transferase DHHC8 is essential for its localization at the plasma membrane and contributes to its efflux function [37]. In the present work, we quantified the mRNA expression of these genes (Table 2), and found that ACAT2 downregulation did not affect their expression. However, we cannot completely exclude their participation in the ABCA1 up-regulation, since only the mRNA expression was evaluated.

The liver is the major organ for HDL synthesis (by lipidation of apoA-I through ABCA1) as shown by the study of liver-specific ABCA1 knockout mice [38]. Whether ACAT2 disruption in mice would have an impact on HDL metabolism was unclear, since previous studies have shown contrasting results on the lipid content in HDL [4], [6], [11]–[14]. In apoE knockout [4] and LDLr knockout [6] mice an increase in HDL cholesterol was observed when ACAT2 was knocked out. However, no changes in HDL lipid composition were observed in different ACAT2^−/−^ mouse genotypes [12], [13], or in mice where ACAT2 was selectively knocked out in the liver or intestine [14]. In the present work we showed that after ASO6 treatment decreased hepatic ACAT2, HDL lipid composition changed only in DOKO mice (increased levels of HDL-UC). Hepatic *Lcat* mRNA was not affected by ASO6 treatment suggesting that the HDL-UC increase is not mediated by a reduction in the levels of this enzyme.

HDL lipoproteins are not just simple lipid carriers, but are a complex and heterogeneous class of particles, differing in physical and chemical properties, protein and lipid composition, metabolism and functions [39]. Treatment of WT mice with ASO6 revealed the appearance of a new peak in the serum lipoprotein profile, which elutes between the typical peak for LDL and HDL particles. We demonstrated that these newly generated lipoproteins are large HDL particles enriched in apoA-I and apoE. Thus, reduction of hepatic ACAT2 activity does not change plasma lipid levels, but rather leads to a redistribution of lipids within the different lipoprotein particles. The newly generated particle appeared independently of the dietary conditions, as was the increase in hepatic ABCA1 expression. This effect could not be observed from the lipid profile of DOKO mice. The big peak generated by the increase in HDL-UC could have possibly masked the peak of the new generated particles for this mouse genotype. Interestingly, large apoE-rich HDL particles seem to be associated with increased hepatic ABCA1 protein expression. This phenomenon has been observed in mice treated with the LXR agonist T090137 [40], in mice overexpressing hepatic ABCA1 [41], and in transgenic mice expressing human NPC1L1in the liver [42]. Lipid poor apoE can directly interact with ABCA1 and be lapidated [43]. This process generates particles with a preβ-migration, and a diameter ranging between 9 and 15 nm [43]. By 2D-gel electrophoresis analysis, we found that ASO6 treatment increased the amount of apoE-containing HDL in both WT and LXR DOKO mice, and that the apoE-containing HDL formed show a progressive shift of migration towards preβ position and an estimated size between 12 and 15 nm. This effect was independent of the dietary challenges.

We also investigated if the changes in HDL metabolism secondary to hepatic ACAT2 knock down resulted in improved HDL functionality. Thus, we measured the capacity of serum to promote cholesterol efflux. This parameter and the different mechanisms involved in cholesterol efflux have been shown to be strongly dependent on the different HDL sub-classes present in the circulation [26], [44]. Indeed, we observed that only when the apoA-I containing particle distribution was modified by ASO6 treatment, serum cholesterol efflux capacity was increased. Importantly, we showed that changes in the efflux pathways involved reflected the changes in preβ- and α-HDL subclasses. These effects were observed only when the western type diet was started prior to the ASO6 treatment, but not when diet and treatment were started concomitantly. In our study we found that serum CEC is positively correlated with both serum and HDL PL content (Figure S3). When the diet was started concomitantly with the treatment ASO6 did not alter the serum PL content, conversely to what observed when the diet was started 4 weeks before the ASO6 treatment. This effect may partly explain the lack of increased serum CEC we observed when the diet was started concomitantly with the ASO6 treatment. It has been shown previously that cellular cholesterol efflux is strongly dependent on the phospholipid content of the extracellular acceptor [32], [45], [46].

In the hepatocytes cholesterol can be esterified by ACAT2 and packed into VLDL, or fluxed into nascent HDL or converted into bile acids. In the present study we also demonstrated that hepatic ACAT2 knock down led to an increased bile acid synthesis, as shown by the higher levels of 7α-hydroxy-4-cholesten-3-one in the liver of the ASO6 treated mice.

Another important finding in this study is the unexpected lack of LXR activation in response to the down-regulation of hepatic ACAT2 activity. No evident changes in mRNA abundance of the classical LXR target genes were observed upon ASO6 treatment. Conversely, decreased hepatic cholesterol esterification in mice led to a down-regulation of the SREBP2 transcriptional pathway since the abundance of *Ldlr*, *Pcsk9*, *Hmgc* reductase and synthase mRNA were decreased. However, this was only evident when the liver was preloaded with dietary cholesterol before down-regulation of ACAT2 activity, since no effects on SREBP2 target genes were observed when ASO6 treatment was started concomitantly with dietary cholesterol challenge. In this latter experimental condition treatment of WT mice with ASO6, the levels of hepatic UC were reduced, and this difference may explain some of the discrepancies observed between the 4-weeks and 0-weeks experiment.

In LXR DOKO compared to WT mice, ACAT2 down-regulation was less effective in reducing hepatic CE in both experiments. For the first time, we have shown here that *Tgh1* and *Tgh2* expression is extremely low in LXRα/β double knockout mice. Since carboxylesterases can also catalyse the hydrolysis of CE, the reduced expression of *Tgh1* and *Tgh2* observed in LXR DOKO may explain the elevated CE content in the livers of LXR DOKO mice, as well as the failure to mobilize it by hydrolysis upon reduction of cholesterol esterification by ASO6 treatment.

A limitation of our study can be represented by the fact that we only investigated liver lipid storage/synthesis, hepatic conversion of cholesterol into bile acids and HDL formation process. Two other important pathways of the hepatic lipid metabolism (i.e. VLDL production and biliary cholesterol secretion) have not been evaluated even if they are known to play a role in HDL metabolism. However the effect of ACAT2 deletion on these pathways has been previously studied. It has been shown that the total apoB accumulation rate in liver perfusate from ACAT2 KO mice was not different from that of their respective littermates [47], and ASO6 treatment led to a reduction of biliary cholesterol secretion in apoB100only-LDLr^−/−^ mice [13].

It is known that ASOs can present non-target effect. In the present work we did not test the effect of ASO6 treatment in ACAT2 knock out (KO) mice to rule out that possibility. Nevertheless, we ran a third experiment (data not shown) feeding ACAT2 KO animals and their respective littermates for 6 weeks with the same western type of diet used in the ASOs experiments. ACAT2 KO showed a similar phenotype to that we observed by ASO6 treatment. Compared to their littermates, ACAT2 KO mice showed double the expression of ABCA1 protein in the hepatic liver membranes together with the appearance of the new HDL lipoprotein particle peaks in the lipid profile.

In conclusion, the use of anti-sense oligonucleotide targeted to hepatic ACAT2 revealed a new pathway by which the liver may contribute to HDL metabolism in mice. ACAT2 seems to be an important hepatocyte player that influences intracellular cholesterol fluxes either into VLDL lipoproteins or into HDL particles, the latter via the up-regulated ABCA1 transporter. The relevance of these findings for the human condition does not seem to be futile. A negative correlation between the hepatic ACAT2 activity and the plasma levels of HDL cholesterol and apoA-I was described in normolipidemic non-obese Chinese patients [48], and a functional variant of the *SOAT2* gene was identified as an independent genetic determinant of plasma HDL cholesterol levels in the LCAS and TexGen cohorts [49]. Thus *SOAT2* has been listed among those genes that influence HDL plasma level in humans [50]. Also, it is worth to remember that treatment with ASO6 generates a condition in mice similar to that in humans, where a low hepatic activity of ACAT2 [51] is present together with elevated intestinal ACAT2 activity [52]. Mice and non-human primates have high hepatic ACAT2 activity [3]. However, the lack of plasma CETP activity [53] or the high rate of hydrolysis of triglyceride rich lipoproteins positively influences the genesis of HDL respectively in these two species. In humans, low hepatic ACAT2 activity may be needed to keep up the lipidation of apoAI. Considering the role of HDL for innate immunity [54], [55], a low hepatic ACAT2 activity may have been a strong evolutionary advantage.

## Supporting Information

Figure S1 **Efflux mechanisms involved in serum cholesterol efflux capacity (CEC).** Cells were radiolabeled with [^3^H]-cholesterol for 24 h, equilibrated in a BSA-containing medium for 18 h and exposed for 4 h to 1% serum from WT (left panels) and LXR DOKO (right panels) mice treated with ASOctrl or ASO6. SR-BI-mediated efflux (A) was assessed in Fu5AH rat hepatoma cells; aqueous diffusion-mediated efflux (B) was assessed in J774 macrophages; ABCA1-mediated efflux (C) was assessed in J774 macrophages: ABCA1 contribution was calculated as the difference between the efflux determined in J774 cells treated with cpt-cAMP 0.3 mM or grown under basal condition. Mean and SEM are shown. Mann Whitney test, *p<0.05; **p<0.01.
(TIF)

Figure S2 **Effect of hepatic ACAT2 down regulation on LDLr and SR-B expression in the liver.** Liver membrane proteins were pooled group-wise, loaded, and separated on Tris-Acetate Gels. After transfer onto nitrocellulose membrane, samples were incubated with anti-mouse LDLr or SR-BI antibody. LDLr band (≈ 140 kD), and SR-BI bands (≈ 75 kD free and ≈50 kD glycosylated form) were detected by chemiluminescence, and signals were plotted by μg-loaded protein. The slope of the curves was calculated by method of least square, and the slope of the ASOctrl group was set equal to 100%. Hepatic *Ldlr* and *Srb1* mRNA were quantified by real-time RT-PCR. Data were standardized for *Tfiib* mRNA expression, and normalized to WT Ctrl in each experiment. mRNA data are expressed as average ± SEM (n = 6–8). Mann Whitney test, * p< 0.05.
(TIF)

Figure S3 **Correlation between serum cholesterol efflux capacity, serum and HDL phospholipids.**
(TIF)

Table S1 **Sequences of the mouse primers.**
(DOCX)
