# Supplementary figures and images for: Hepatic ACAT2 Knock Down Increases ABCA1 and Modifies HDL Metabolism in Mice
Source: PLoS One. 2014 Apr 2;9(4):e93552. doi: 10.1371/journal.pone.0093552 (PMC3973598; doi:10.1371/journal.pone.0093552)

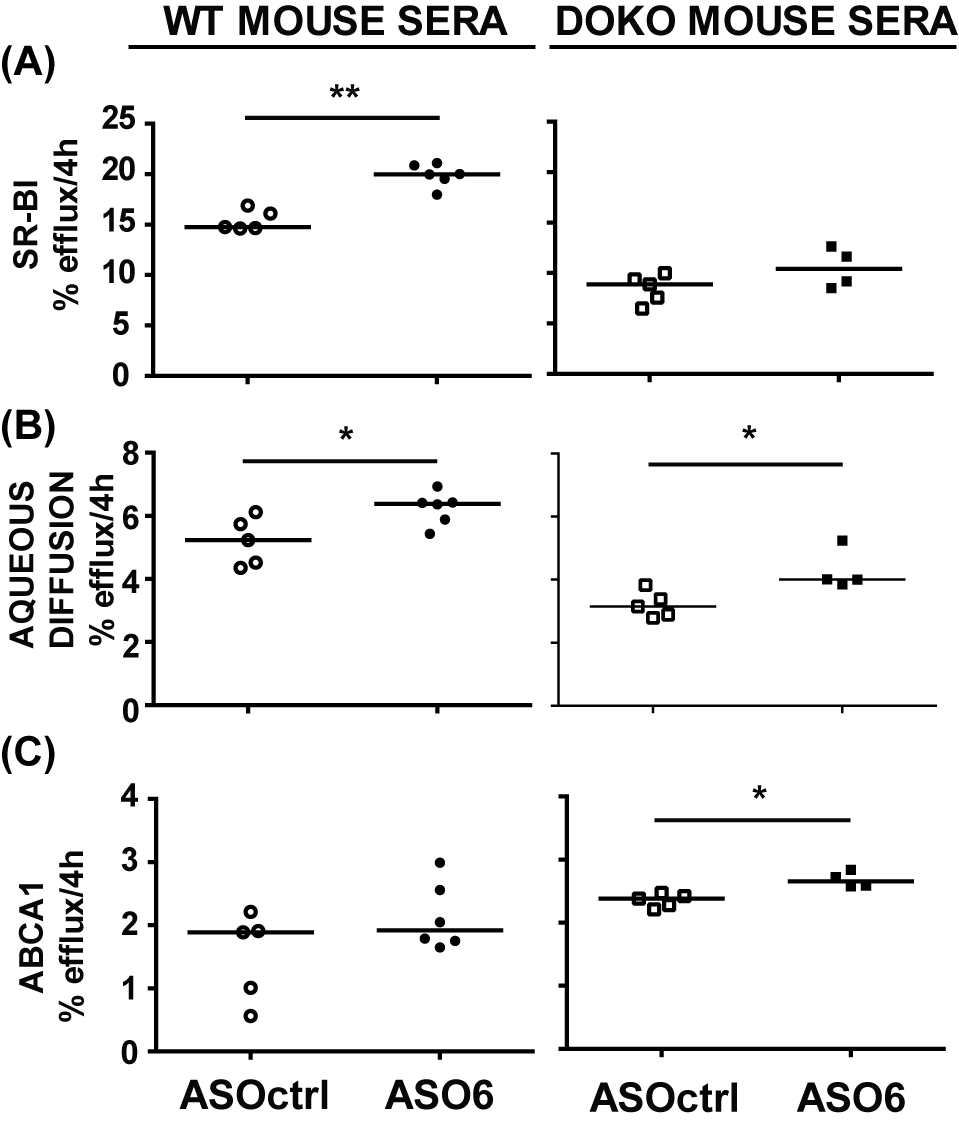

Supplement: Figure S1 — Efflux mechanisms involved in serum cholesterol efflux capacity (CEC). Cells were radiolabeled with [3H]-cholesterol for 24 h, equilibrated in a BSA-containing medium for 18 h and exposed for 4 h to 1% serum from WT (left panels) and LXR DOKO (right panels) mice treated with ASOctrl or ASO6. SR-BI-mediated efflux (A) was assessed in Fu5AH rat hepatoma cells; aqueous diffusion-mediated efflux (B) was assessed in J774 macrophages; ABCA1-mediated efflux (C) was assessed in J774 macrophages: ABCA1 contribution was calculated as the difference between the efflux determined in J774 cells treated with cpt-cAMP 0.3 mM or grown under basal condition. Mean and SEM are shown. Mann Whitney test, *p<0.05; **p<0.01. (TIF) [file pone.0093552.s001.tif]

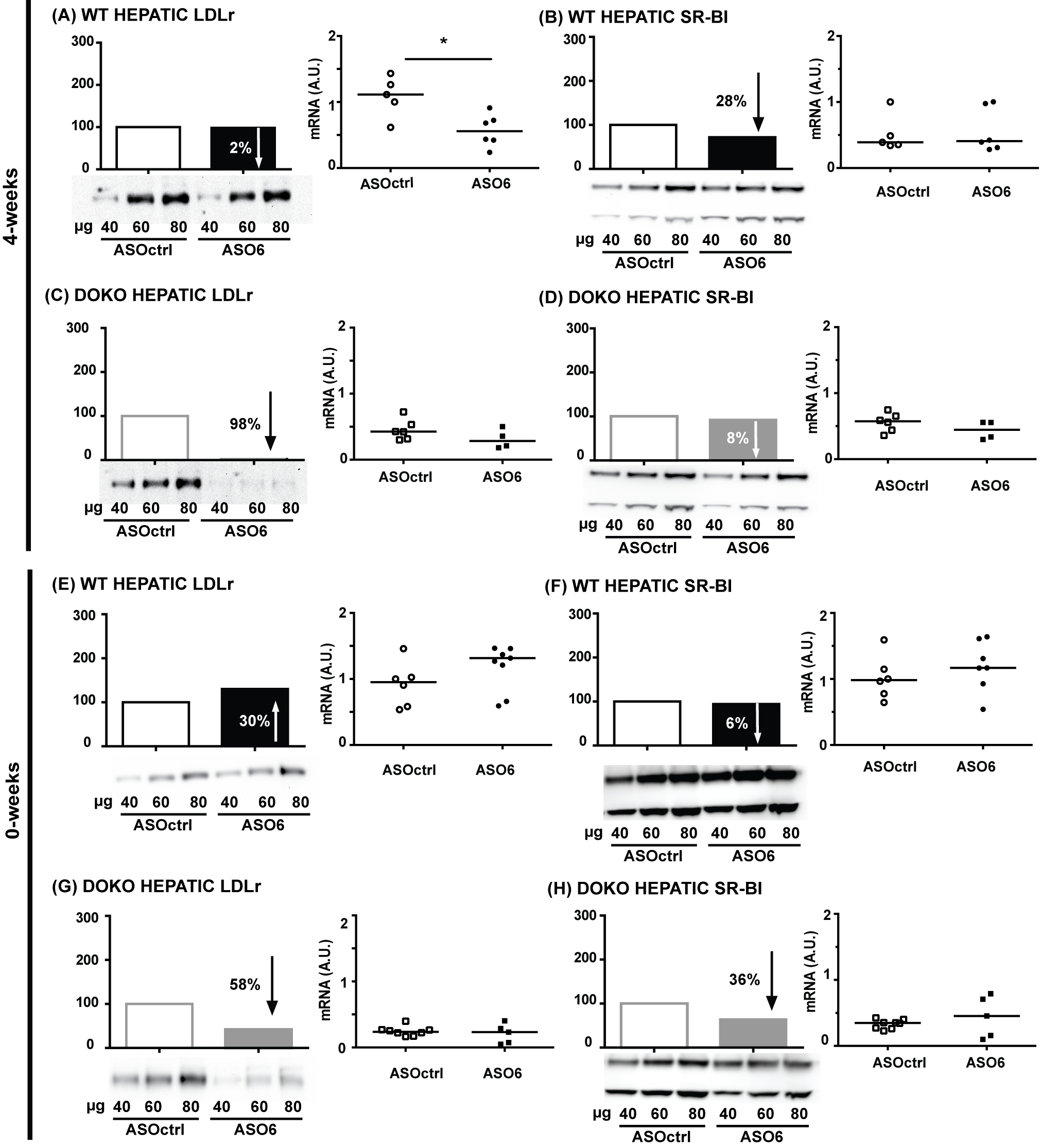

Supplement: Figure S2 — Effect of hepatic ACAT2 down regulation on LDLr and SR-B expression in the liver. Liver membrane proteins were pooled group-wise, loaded, and separated on Tris-Acetate Gels. After transfer onto nitrocellulose membrane, samples were incubated with anti-mouse LDLr or SR-BI antibody. LDLr band (≈ 140 kD), and SR-BI bands (≈ 75 kD free and ≈50 kD glycosylated form) were detected by chemiluminescence, and signals were plotted by μg-loaded protein. The slope of the curves was calculated by method of least square, and the slope of the ASOctrl group was set equal to 100%. Hepatic Ldlr and Srb1 mRNA were quantified by real-time RT-PCR. Data were standardized for Tfiib mRNA expression, and normalized to WT Ctrl in each experiment. mRNA data are expressed as average ± SEM (n = 6–8). Mann Whitney test, * p< 0.05. (TIF) [file pone.0093552.s002.tif]

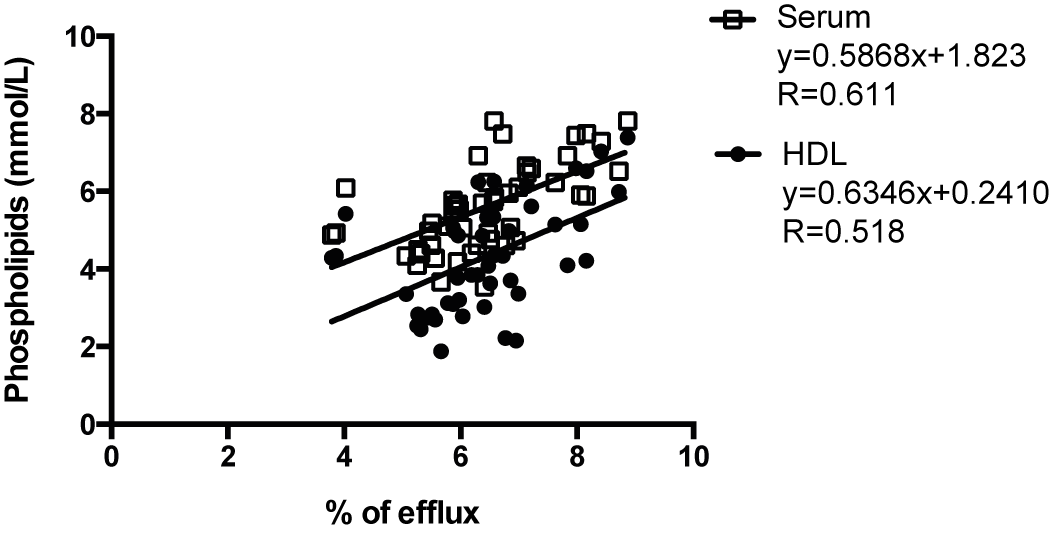

Supplement: Figure S3 — Correlation between serum cholesterol efflux capacity, serum and HDL phospholipids. (TIF) [file pone.0093552.s003.tif]
