# Supplementary material for: Hepatic ACAT2 Knock Down Increases ABCA1 and Modifies HDL Metabolism in Mice
Source: PLoS One. 2014 Apr 2;9(4):e93552. doi: 10.1371/journal.pone.0093552 (PMC3973598; doi:10.1371/journal.pone.0093552)
Supplement: Table S1 — Sequences of the mouse primers. (DOCX) [file pone.0093552.s004.docx]

| **Gene** | **Forward** | **Reverse** |
| --- | --- | --- |
|  |  |  |
| ***Srebp2*** | GCG TTC TGG AGA CCA TGG A | ACA AAG TTG CTC TGA AAA CAA ATC |
| ***Hmgcr*** | TGA TTG GAG TTG GCA CCA T | TGG CCA ACA CTG ACA TGC |
| ***Hmgcs*** | CTC TGT CTA TGG TTC CCT GGC T | CCC AAT CCT CTT CCC TGC C |
| ***Pcsk9*** | CAG GGC TGG AAT GCA AAA TC | GCC ACA GTG ACC TGC TCT GA |
| ***Lcat*** | GTG GTT CCA TCA AGG CCA TG | TGC CCT GGT TGT CAC CTG A |
| ***Lipc*** | AGA GAC GCA GCA AAG AAT GAC A | TGG AGG TCA TCC AGA TTT TCG |
| ***Cyp7a1*** | AGC AAC TAA ACA ACC TGC CAG TAC TA | GTC CGG ATA TTC AAG GAT GCA |
| ***Abcg5*** | AAT GCT GTG AAT CTG TTT CCC A | CCA CTT ATG ATA CAG GCC ATC CT |
| ***Abcg8*** | TGC CCA CCT TCC ACA TGT C | ATG AAG CCG GCA GTA AGG TAG A |
| ***Scd1*** | CCG GAG ACC CCT TAG ATC GA | TAG CCT GTA AAA GAT TTC TGC AAA |
| ***Fasn*** | GCT GCG GAA ACT TCA GGA AAT | AGA GAC GTG TCA CTC CTG GAC TT |
| ***Acc1*** | GGA CAG ACT GAT CGC AGA GAA AG | TGG AGA GCC CCA CAC ACA |
| ***Srebp1c*** | GGA GCC ATG GAT TGC ACA TT | GGC CCG GGA AGT CAC TGT |
| ***Tgh1*** | AGA CCT GGA GCT CCG TGA AG | CTG GCC CCA CTC CTG TAA TTT |
| ***Tgh2*** | AGG CCA GAA GGC TGC AGT T | GCT TGT CCT GAG AAC CCT TGA G |
| ***Ctsd*** | ATT TGT TGC AGC CAA GTT TGA TG | AAG ACC GGA AGC ACG TTG TT |
| ***Zdhhc8*** | CGC CGC AAC TAC CGT TAC TT | GTG CGC GCT GAG TGA CAG |
| ***Rab8a*** | AGC GGT TTC GAA CAA TCA CG | TGA TAC CCA TGG CAC CCC T |
|  |  |  |

**Table S1**
